# Barriers and Challenges in Performing Pharmacokinetic Studies to Inform Dosing in the Neonatal Population

**DOI:** 10.3390/pharmacy8010016

**Published:** 2020-02-05

**Authors:** Kate O’Hara, Jennifer H. Martin, Jennifer J. Schneider

**Affiliations:** Discipline of Clinical Pharmacology, School of Medicine, University of Newcastle, Hunter Medical Research Institute Newcastle, Newcastle 2308, Australia; Jenniferh.martin@newcastle.edu.au (J.H.M.); jennifer.schneider@newcastle.edu.au (J.J.S.)

**Keywords:** neonate, pharmacokinetics, translational research, ethics, biomedical, modeling, drug

## Abstract

A number of barriers and challenges must be overcome in order to conduct the pharmacokinetic studies that are urgently needed to inform the selection and dosing of medication in neonates. However, overcoming these barriers can be difficult. This review outlines the common barriers researchers are confronted with, including issues with ethics approval and consent, study design for pharmacokinetic studies and the ability to measure the drug concentrations in the blood samples obtained. Strategies to overcome these challenges are also proposed.

## 1. Introduction

While clinical research to inform drug dosing in neonates was identified as a therapeutic imperative many years ago, progress in this area has been slow [1]. Most neonatal drug doses are still based on extrapolation from adult and paediatric pharmacokinetic studies and dosing information, rather than studies performed in the neonatal population. As a result, a number of drugs have been introduced to the nursery without sufficient research, with disastrous consequences, and many others causing unnecessary morbidity [2,3,4]. This would not be accepted as best practice, or even reasonable practice, in other patient populations [5].

Pharmacokinetic studies play a pivotal role in informing the design of dosage regimens. Performing these studies involves several steps, including gaining funding, the approval of the study by an ethics committee, an appropriate study design, which includes determining the required sample size and study power, blood sampling strategies and the analysis of blood samples. The aim of this review is to identify the barriers to this type of research in the neonatal population and suggest possible solutions.

## 2. Ethical Considerations

### 2.1. Risk Versus Benefit

In 2003, the Food and Drug Administration (FDA) and European Medicines Agency (EMA) convened a panel of experts to examine the use of off-patent drugs in the neonatal population [6,7]. The panel concluded that the judicious use of limited resources in this area remains an urgent public health need, and identified criteria for prioritizing and supporting the decision to perform a study in newborns [6]. The criteria included disease and indication, potential for adverse outcomes, level of evidence for treatment of newborns, lack of information on age-appropriate formulation, drug–drug and drug–disease interactions, drug disposition in newborns, feasibility and methodology, including both analytical considerations and clinical endpoints, and the ethical basis for the study, including addressing benefit versus harm due to exposure to the study drug [6]. These criteria [6] provide a framework that may be used by clinicians and researchers in first deciding and then justifying their decision to perform research in this population [7].

Children are a uniquely vulnerable group and considerations of the level of risk versus benefit differ from those in an adult population. While adults may consent to studies that are risky and not individually beneficial, studies in children and neonates must have minimal risks or the benefits, to both the individual and the community, must outweigh the risks [8]. Furthermore, many standard trial interventions carry a greater risk to neonates [6] due to a lack of information about developmental changes and the relative size of the intervention (e.g., blood or biopsy sampling) required. This consideration of benefit versus risk can be demonstrated by considering the use of analgesics in neonates and children. Providing analgesia is an essential part of good clinical care for all patients, no matter their age. However, as of 2018, the FDA has not approved licensing for any analgesics for use in children less than 6 months old [9]. The use of analgesic drugs in these populations “off label” may be beneficial but may also entail risk, as few data are available to guide dosing. Clinical trials investigating the efficacy, safety and dosing of analgesics across all paediatric age cohorts are needed and are readily justifiable [10], as analgesics meet many of the criteria for the prioritization outlined earlier.

### 2.2. Lack of Expertise

Ethics committees may lack members with neonatal expertise and, therefore, may err towards caution when reviewing neonatal studies. As a result, the number of studies conducted may be limited or studies may be significantly delayed, even those with a minimal impact on patients and their families. The current “standard of care” may also influence the decision to approve a study, even if the current therapies are not based on evidence [11], due to concerns that the proposed interventions differ from standard practice. Health professionals with experience and training in neonatal care, including pharmacists, should be encouraged to pursue opportunities to be involved as members of local ethics committees to address this imbalance.

### 2.3. Consent

The admission of a child to hospital is a stressful time for a family, particularly so with neonates. A parent or guardian is required to consent to neonatal participation for any research study [12]. Parents may struggle with the idea of volunteering their child for research studies during a period of illness. The thought of additional procedures may add to parental concerns regarding research participation. The evidence that informed and voluntary consent can be obtained under these conditions is limited [11], although it has successfully occurred in the past with clinical trials for surfactant, where consent was sought antenatally [13]. There are some limits to parental consent, with parents not able to give consent for their children to participate in as wide a range of studies as an autonomous adult [12]. Parents themselves have also noted concerns about consent, including that both parents should provide consent [14]. Best practice regarding consent and recruitment involves a process of continuous consent in the days and weeks after birth, with repeated discussions with the family, including the opportunity to confirm or withdraw consent. Appropriately credentialed nurses and pharmacists are able to take consent for recruitment into medicinal trials once the physician has undertaken a medical assessment for eligibility [15].

## 3. Development of Pharmacokinetic Modeling Methods

For pharmacokinetic studies which require blood sampling, researchers should consider whether the opportunity exists to use a portion of the blood samples taken for other clinical monitoring. This approach has been used successfully [6,16] and avoids having the neonate undergo additional sampling.

Developing pharmacokinetic models to inform dosing traditionally required several consecutive blood samples to be taken from each patient in order to describe the plasma drug concentration versus time curve. This approach often precluded the modeling of drugs in children, as it was not possible or ethical to take the large number of blood samples that were required from children, especially neonates. The development of population pharmacokinetic modeling approaches has overcome the limitations of traditional pharmacokinetic studies. Population pharmacokinetic models use concentration time points from a number of clinical subjects to determine the pharmacokinetics of drugs in that population and can be designed to use the minimum number of samples possible from each patient [17].

Mixed-effects population pharmacokinetic models may be applied to neonatal pharmacokinetic studies. These models allow the study of variability in drug concentrations in a sample population which represents the population in which the drug will be used clinically. These models use the population as the unit of analysis, rather than the individual. Mixed-effects models differ from other methods as they use a small number of samples from a large number of subjects. This approach allows the data to be described using a mixture of fixed and random effects. Fixed effects predict the average effect of a covariate, such as weight or sex, as a possible explanation of part of the inter-subject variability in a pharmacokinetic parameter, such as clearance. Random effects are used to describe the variability that remains and is not predictable from an average of the relevant fixed effects. The predictable parts of the inter-subject variability can be assigned explanatory covariates such as age, size, sex, temperature or renal function [18]. Mixed-effects models, in particular, are useful for examining the developmental aspects of drug metabolism [19] and are most suitable for use in neonates. Implementing these into clinical practice, however, is another barrier which organisations need help to overcome.

Determining the optimal times for blood sampling also assists in minimising the number of interventions required. D-optimal design can be particularly useful in planning neonatal studies, as it enables the selection of the best sampling times for incorporation into a model. It allows for the refining of the model as more becomes known about how it behaves with different data points from the set [20]. In this way, the number and timing of the samples required to provide the optimal data set for modeling can be determined [21].

While population pharmacokinetic studies reduce the number of blood samples required for each patient, recruiting an appropriate number of participants still remains a problem [6]. For neonates, as only a small number of babies require neonatal intensive care, a study may take a long time to complete if it is conducted at just one centre. One way to overcome this is to develop collaborations across several centres, improving the rate and amount of data that can be collected to build pharmacokinetic models [7,22]. However, ethics and governance processes for multi-site studies need to be streamlined at an organizational level to make this feasible for clinicians and researchers to manage.

Multisite studies for population pharmacokinetics are also challenging due to the variation in drug dosing used across different sites [11]. Different doses present an additional variable which increases the number of participants and samples required to develop the model. Although a number of dosing resources exist, they are based on limited evidence and are often overruled by individual clinical experience. The first step in successfully completing this type of research is standardizing the drug dosing across a number of sites, which will allow for participant recruitment at a faster rate than could be achieved in a single unit and the development of a more detailed model.

## 4. Measuring Plasma Drug Concentrations

Analysis of neonatal samples requires techniques that are capable of measuring low drug concentrations in small volume samples. A limitation to neonatal pharmacokinetic studies is that commonly available high performance liquid chromatography with ultraviolet detection (HPLC-UV) systems are generally unable to achieve the required sensitivity in small volume samples. The emergence of new HPLC systems with triple quadrupole mass spectrometry detection (HPLC-MS/MS) has greatly increased the sensitivity and significantly decreased the volume of plasma or blood required for the analysis [7]. Sample volumes of 10 to 100 μL may be analysed using this technology, compared to the 1 to 2 mL samples required with HPLC-UV systems. It is recommended that pharmacokinetic studies limit sampling to a blood volume of 3 mL/kg [21]. While neonates were unable to provide the large number or volume of samples required for HPLC-UV analysis, the volumes required with HPLC-MS/MS now make pharmacokinetic studies feasible. HPLC-MS/MS also requires low injection volumes, allowing for re-analysis, if required, without re-sampling. The availability of assays requiring very small blood volumes is an important advance in neonatal research. Currently, access to this equipment and the skilled personnel to perform the analyses may be a barrier. However, with the increasing uptake of this technology by analytical laboratories, access should become easier in the near future.

An exciting development in the field of blood sample analysis and neonatal pharmacokinetic studies is the availability of dried blood spot (DBS) sampling and volumetric absorptive sampling techniques, such as the Mitra^®^ device. DBS has been used successfully in neonatal care for many decades in newborn screening. The usefulness of DBS sampling for pharmacokinetic analysis has been demonstrated in recent studies [23,24]. Samples may be obtained by heel prick and only a very small drop of blood (often 10–20 μL) is required. These sampling techniques have a minimal impact on patients, require a small volume of blood, and reduce the difficulties related to the handling of larger volume blood samples, which may require refrigeration and special shipping. DBS are usually very stable and may be transported and stored at ambient temperatures. This feature would enable centres without ready access to analytical facilities to easily send samples to another centre for analysis. A combination of these low volume sampling techniques with highly sensitive assays presents the best approach to developing neonatal pharmacokinetic studies.

## 5. Solutions

The solutions to these complicated issues are multifactorial and dependent on wide-scale change and increased organizational support for neonatal research, particularly around the streamlining of ethics and governance procedures for multi-site studies. The increased representation of multidisciplinary neonatal clinicians to advocate on ethics committees, hospital leadership committees, funding organisations and regulatory bodies could help raise the profile of this research area. However, it is well known that clinician time for additional service outside of clinical care is unfunded and burdensome. Support for clinical staff to have the dedicated time to conduct translational research, within their current workload, would improve the completion rate of this research.

Increasing the neonatal expertise on ethics committees will reduce the number of concerns raised when neonatal research proposals are submitted. Comprehensive international guidelines [6,9,11,25], addressing areas such as appropriate blood sampling and consent for neonates, are available and could be adopted by ethics committees worldwide. Increasing the use of antenatal consent from parents may help in improving participation rates by allowing more time for discussions about being involved in the study.

The development of low volume plasma/blood analysis techniques improves the feasibility of conducting this type of research. These techniques, combined with population pharmacokinetic modeling, allow researchers to use blood left over from clinical samples, resulting in negligible effects on the research participants.

Further research into drug concentration analysis techniques using low plasma volume or DBS technology will expand the number of drugs than can be studied in neonates. Once access to the equipment is established, this research will have low running costs beyond staff time. The fast throughput of the methodologies allows for large numbers of samples to be processed in a single session.

Once the research is completed, an efficient approach is required to quickly translate the findings to practice and update medicine formularies [7]. Research networks should work closely with local formulary committees and governance bodies to develop evidence-based dosing information that can be used for patients. Pharmacists, as part of their clinical practice, should consider opportunities for the publication of data collected during clinical care to help inform the prioritization and design of clinical studies. Neonatal pharmacy should continue to be promoted as a specialist option to allow pharmacists to bring their unique skills to improving the use of medicines in this population.

## 6. Summary

Advances in technology and pharmacokinetic modeling techniques are now making research in neonatal pharmacokinetics and dosing feasible. To utilize these advances, large-scale international pharmacokinetic studies in neonates need to be established. Building the required collaborations could begin by sharing clinical experiences with other clinicians and publishing preliminary observations and data. Mutidisciplinary advocacy to streamline the barriers in the ethics process and governance in healthcare organisations is required. Encouraging health professionals working in neonatal care to be involved in ethics committees and other related organisations will help further this research. Strong advocacy and good clinical and research leadership are increasingly recognized as drivers of change in health care. Advocating for clinicians to have dedicated research time as part of their workload would also be a positive step forward.
